# Anti-antimicrobial Peptides: FOLDING-MEDIATED HOST DEFENSE ANTAGONISTS*[Fn FN2]

**DOI:** 10.1074/jbc.M113.459560

**Published:** 2013-06-04

**Authors:** Lloyd Ryan, Baptiste Lamarre, Ting Diu, Jascindra Ravi, Peter J. Judge, Adam Temple, Matthew Carr, Eleonora Cerasoli, Bo Su, Howard F. Jenkinson, Glenn Martyna, Jason Crain, Anthony Watts, Maxim G. Ryadnov

**Affiliations:** From the ‡ National Physical Laboratory, Teddington, Middlesex TW11 0WL, United Kingdom,; the § Division of Biomedical Sciences, St. George's University of London, London SW17 0RE, United Kingdom,; the ¶ School of Oral and Dental Sciences, University of Bristol, Bristol BS1 2LY, United Kingdom,; the ‖ Department of Biochemistry, University of Oxford, Parks Road, Oxford OX1 3QU, United Kingdom,; the ** School of Physics and Astronomy, University of Edinburgh, Edinburgh EH9 3JZ, Scotland, United Kingdom, and; the ‡‡ IBM T. J. Watson Research Center, Yorktown Heights, New York 10598

**Keywords:** Antimicrobial Peptides, Membrane Biophysics, Microscopy, Molecular Dynamics, Nuclear Magnetic Resonance, Protein Design, Protein Folding

## Abstract

Antimicrobial or host defense peptides are innate immune regulators found in all multicellular organisms. Many of them fold into membrane-bound α-helices and function by causing cell wall disruption in microorganisms. Herein we probe the possibility and functional implications of antimicrobial antagonism mediated by complementary coiled-coil interactions between antimicrobial peptides and *de novo* designed antagonists: anti-antimicrobial peptides. Using sequences from native helical families such as cathelicidins, cecropins, and magainins we demonstrate that designed antagonists can co-fold with antimicrobial peptides into functionally inert helical oligomers. The properties and function of the resulting assemblies were studied in solution, membrane environments, and in bacterial culture by a combination of chiroptical and solid-state NMR spectroscopies, microscopy, bioassays, and molecular dynamics simulations. The findings offer a molecular rationale for anti-antimicrobial responses with potential implications for antimicrobial resistance.

## Teaser

**Background:** Direct antagonists of native antimicrobial peptide (AMP) sequences are unknown.

**Results:** Complementary antagonistic sequences can co-fold with AMPs into functionally inert assemblies.

**Conclusion:** Antagonists act as anti-AMPs.

**Significance:** The findings offer a molecular rationale for anti-AMP responses with potential implications for antimicrobial resistance.

## Introduction

Intermolecular recognition by peptides is a critical aspect of regulatory processes at cellular interfaces (1). Native systems ranging from viral fusion proteins (2) and SNARE complexes (3) to membrane skeletal networks (4) and harbor domains of enteric pathogens (5) rely on specific α-helical coiled coils to maintain interfacial contacts in a highly cooperative manner. Amphipathic helical monomers can be designed to incorporate alternative interfaces into such assemblies, thereby disrupting regulatory processes (6). By contrast, amphipathic antimicrobial helices bind to microbial membranes without *a priori* requirements for specific assemblies (7). Given their sequence similarities with coiled coils, antimicrobial peptides may be challenged by co-assembly with antagonistic helices, a proposition that has so far been unexplored. Thus, the overall aim of this study is to explore the hypothesis that antimicrobial activity in peptides can be effectively neutralized by the formation of inert coiled-coil complexes.

**FIGURE 1.**
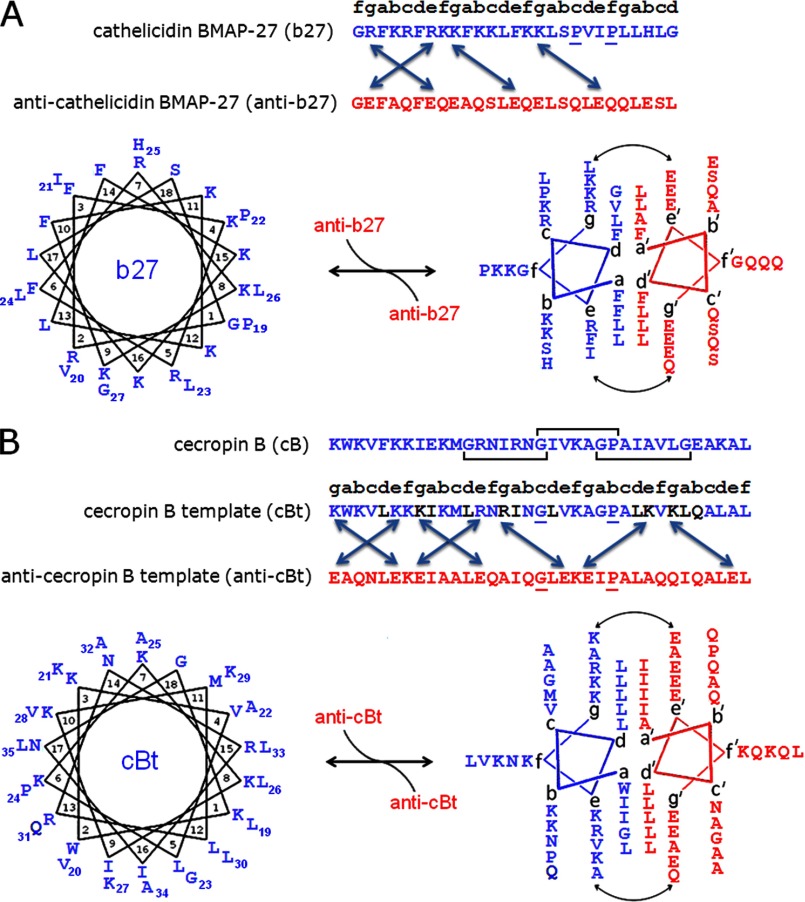
**Peptide design.** *A*, cathelicidin type: bovine myeloid antimicrobial peptide-27 (b27) and anti-b27. *B*, cecropin type: native cecropin B (cB), cecropin B template (cBt), and anti-cecropin B template (anti-cBt). Linear sequences (*top*) and configured onto helical wheels (*bottom*) are monomeric with 3.6 residues (*left*) and coiled-coil with 3.5 residues (*right*) per turn. Antimicrobial peptides and antagonists are shown in *blue* and *red*, respectively. Mutations in cBt are in *black*, and key residues in helix-disrupting motifs are *underlined*. Three overlapping G(*X*)_*n*_G motifs are highlighted by *horizontal brackets. Double-headed arrows* denote *e*lectrostatic *e-g*′ interactions.

Coiled-coil sequences show heptad repeats of hydrophobic and polar residues (usually designated *abcdefg*), in which *a* and *d* form hydrophobic interfaces (8). To form contiguous interfaces the *a*/*d* patterns of corresponding helices must be in register, which is prevented by the average spacing of hydrophobic residues along a coiled-coil sequence being 3.5 residues. This is less than one complete turn (3.6 residues) of a monomeric α-helix (9). To rectify this discrepancy *a*/*d* patterns impose a slight left-handed twist enabling left-handed helix-crossing angles in the coiled-coil bundle, which permits, but does not guarantee, stable coiled coils. Further stabilization is required through complementary electrostatic interactions at *e* and *g* sites of successive heptads between partner strands (*i.e. g-e*′ interactions: *g* of one heptad and *e*′ of the following heptad on the other helix) (Fig. 1) (8, 9). Therefore, in assigning coiled-coil patterns in antimicrobial sequences it is necessary to consider hydrophobic *a*/*d* pairs in conjunction with charged *e*/*g* pairs.

Antimicrobial peptides (AMPs)^2^ are cationic amphipathic structures that fold and oligomerize in anionic phospholipid membranes (7). By this convention, antagonistic sequences are anionic amphipathic helices that remain unfolded in solution and in membrane environments but fold upon binding to cationic AMPs (Fig. 1). The strength and stability of this binding depend on helical propensity and persistence length. An antimicrobial sequence can comprise up to 50 amino acid residues (7, 10; see also antimicrobial sequences database at the bbcm Web site at the University of Trieste, Italy), and most helical AMPs contain 20–35 residues which should be sufficient for coiled-coil formation (11). However, and importantly, native antimicrobial helices often incorporate helix-disrupting motifs to facilitate membrane insertion. Common examples include glycine zipper G(*X*)_*n*_G motifs, where *X* is any residue and *n* = 3–6 (12, 13). Length correlations between these motifs and antimicrobial peptides are not obvious. For instance, bombinins prefer *n* = 3 as do membrane proteins that incorporate glycine zippers for transmembrane helix dimerization, whereas cecropins, defensins, and magainins tend to have variable *n*, a strategy also used by proteins to specify phosphate binding and domain folding (14–19). Given these dependences and preferences for longer coiled coils in membrane proteins (2–5) it was appropriate to probe one sequence having a relatively long and uninterrupted helical stretch and another longer sequence containing G(*X*)_*n*_G motifs. A cathelicidin, bovine myeloid antimicrobial peptide-27 (b27) (20), and cecropin B (cB), originally isolated from the cecropia moth *Hyalophora cecropia* (21) met the requirements (Fig. 1).

## EXPERIMENTAL PROCEDURES

### 

#### 

##### Peptide Synthesis

All peptides were synthesized on a Liberty microwave peptide synthesizer (CEM Corporation) using standard solid phase Fmoc (*N*-(9-fluorenyl)methoxycarbonyl) protocols on Rink amide-4-methylbenzhydrylamine resins with HCTU/DIPEA as coupling reagents. Peptides were purified by semipreparative RP-HPLC on a JASCO HPLC system (model PU-980; Tokyo, Japan) and confirmed by MALDI-TOF mass spectrometry (Bruker Daltonics) with α-cyano-4-hydroxycinnamic acid as the matrix.

MS [M+H]^+^: b27, *m*/*z* 3282.2 (calc.), 3283.2 (found); anti-b27, 3138.3 (calc.), 3139.3 (found); cB, *m*/*z* 3834.5 (calc.), 3836.0 (found); cBt, *m*/*z* 3968.1 (calc.), 3969.1 (found); anti-cBt, *m*/*z* 3843.4 (calc.), 3843.4 (found); m2, *m*/*z* 2465.9 (calc.), 2467.0 (found); m2t, *m*/*z* 2526.1 (calc.), 2526.1 (found); m2t2, *m*/*z* 2555.3 (calc.), 2556.2 (found); anti-m2, *m*/*z* 2529.8 (calc.), 2529.8 (found); anti-m2t2, *m*/*z* 2560.9 (calc.), 2562.0 (found). [M+Na]^+^ and [M+K]^+^ were also found.

##### High Performance Liquid Chromatography

Analytical and semipreparative gradient RP-HPLC was performed on a JASCO HPLC system using Vydac C18 analytical (5 μm) and semipreparative (5 μm) columns. Both analytical and semipreparative runs used a 10–60% B gradient over 50 min at 1 ml/min and 4.5 ml/min, respectively, with detection at 230 and 220 nm. Buffer A was 5% and buffer B was 95% aqueous CH_3_CN, 0.1% TFA.

##### Lipid Vesicle Preparation

The lipids, 1,2-dilauroylphosphatidylcholine (DLPC) and 1,2-dilauroyl-*sn*-glycero-3-phospho-(1′-rac-glycerol) (DLPG), 75%/25% (w:w) used for liposome construction were from Avanti Polar Lipids. The lipids were weighted up, dissolved in chloroform-methanol (2:1, v:v), dried under a nitrogen stream, and placed under vacuum overnight. The resulting film was hydrated to 10 mg/ml total lipid concentration in 10 mm phosphate buffer, pH 7.4. The suspension was then extensively vortexed, sonicated (30 °C), and extruded using a hand-held extruder (Avanti Polar Lipids) (15 times, polycarbonate filter, 0.05 μm) to give a clear solution containing small unilamellar vesicles, which were analyzed (50 nm) by photon correlation spectroscopy.

##### Photon Correlation Spectroscopy

Vesicles were resuspended to a final concentration of 1 mg/ml and were analyzed on a Zetasizer Nano (ZEN3600, Malvern Instruments, Worcestershire, UK). Dynamic light scattering batch measurements were carried out in a low volume disposable cuvette at 25 °C. Hydrodynamic radii were obtained through the fitting of autocorrelation data using the manufacture's software, Dispersion Technology Software (DTS version 5.10).

##### Circular Dichroism Spectroscopy

All CD spectra were recorded on a JASCO J-810 spectropolarimeter fitted with a Peltier temperature controller. All measurements were taken in ellipticities in millidegrees and converted to molar ellipticities ([θ], deg cm^2^ dmol^−1^) by normalizing for the concentration of peptide bonds. Aqueous peptide solutions (300 μl, at a given concentration) were prepared in filtered (0.22 μm) 10 mm phosphate buffer, pH 7.4. CD spectra recorded in the presence of synthetic membranes are for lipid:peptide molar ratio of 100:1. CD titrations were performed at 20 °C by titrating anti-cBt (177.5 μm) into cBt (30 μm) to achieve anti-cBt/cBt molar ratios from 0.1 to 2.

##### Linear Dichroism Spectroscopy

Solution-phase flow linear dichroism spectroscopy was performed on a JASCO-810 spectropolarimeter using a photo elastic modulator 1/2 wave plate, and a micro-volume quartz Couette flow cell with ∼0.25 mm annular gap and quartz capillaries (all from Kromatec Ltd.). Molecular alignment was achieved through the constant flow of the sample solution between two coaxial cylinders: a stationary quartz rod and a rotating cylindrical capillary. Linear dichroism (LD) spectra were acquired with laminar flow obtained by maintaining the rotation speed at 3000 rpm and processed by subtracting nonrotating base-line spectra. LD spectra recorded in the presence of synthetic membranes, DLPC and DLPC:DLPG (3:1), were prepared at a lipid:peptide molar ratio of 100:1 (2 mm total lipid, 20 μm peptide).

##### FTIR Spectroscopy

All FTIR spectra were collected using a Tensor-37 series FTIR spectrophotometer with a BioATR II unit (Bruker Optics) as the sampling platform with a photovoltaic MCT detector and a Bruker Optics work station, which was equipped with OPUS software. Aqueous samples of very low volume (15 μl, 50–130 μm) were placed in a circular sampling area of radius 2 mm with a path length of 6 μm. This multireflection ATR accessory is based on a dual crystal technology, which has an upper silicon crystal and a hemispherical zinc-selenide (ZnSe) lower crystal that does not come into contact with the sample. The temperature of the sample was maintained at 20 °C by means of flow connectors to a circulating water bath. This accessory was purged continuously throughout the experiment with dry nitrogen via telescopic inserts that seals the optical path inside the spectrometer sample compartment. All FTIR spectra were collected with resolution 4 cm^−1^, scanner velocity 20 kHz, 256 scans, phase resolution 32, and zero filling factor 4.

##### Analytical Ultracentrifugation

Sedimentation equilibrium experiments for anti-cBt and cBt peptides and their equimolar mixture were carried out at 20 °C in a Beckman Optima XL-I analytical ultracentrifuge fitted with absorbance and interference optics. Sedimentation equilibrium curves were measured by interference optics in the 1.2-cm path length cells. 100-μl samples of 100 μm peptides, individually and as equimolar mixtures buffered to pH 7 with 20 mm potassium phosphate, were used. Buffer density was taken as 1.0007 mg/ml, and samples were equilibrated for 24 h at rotor speeds of 30,000, 37,000 and 50,000 rpm. The data were fitted using routines in Origin (Origin Lab). The average partial specific volumes for the peptides were calculated from the amino acid sequences and were 0.73 ml/mg for all samples.

##### Isothermal Titration Calorimetry

Measurements were obtained using a Microcal VP-isothermal titration calorimeter and a calorimetric cell (initial volume 1.46 ml) with a 260-s equilibration time and a 120-s initial delay after each addition. The titrations were performed at 20 °C with stirring until no further enthalpy changes were observed. Binding isotherms were recorded for cBt (30 μm) following the injection of anti-cBt (2 μl aliquots, 1.4 mm) into the cell. The observed heats were corrected for dilution effects by titrating peptide solutions, as appropriate, into the buffer and using the heat of last injections due to negligible differences between the first and last injections. All the data were corrected for the volume of the added titrant and analyzed by proprietary software (Microcal Origin 7) using a one-site binding model to allow for the determination of association constants (*K*_*a*_), changes in enthalpy (Δ*H*) and enthropy (Δ*S*). Each experiment was performed in triplicate.

##### Solid-state NMR

DMPC-d54 and DMPG were purchased from Avanti Polar Lipids and used without further purification. A 3:1 (molar ratio) of DMPC:DMPG (8 mg of total lipid) was solubilized in a 2:1 mixture (v/v) of chloroform and methanol and dried under a stream of N_2_ gas to generate a thin film. The lipid film was subsequently rehydrated with 20 mm HEPES buffer (containing deuterium-depleted H_2_O and peptide) at pH 7 to a final volume of 30 μl. 1 mg/ml stock solutions of cecropin mutant and blocker peptides were prepared in deuterium-depleted HEPES buffer at pH 7 and were added to the hydrated lipid to give a final total lipid:peptide molar ratio of 25:1. In samples in which the cecropin mutant and blocker were both present, the individual stock peptide solutions were combined before being diluted and added to the lipid film for rehydration. Static ssNMR experiments were carried out on a Varian Infinityplus 500-MHz spectrometer equipped with a 4-mm MAS HXY probe at 30 °C. All samples were prepared with total lipid concentrations of 10 mg/ml in 20 mm pH 7 HEPES buffer. Static ^2^H ssNMR spectra were acquired at 76.8 MHz using a quadrupolar echo sequence with a recycle delay of 0.5 s. 200,000 scans were collected, and ^2^H pulse lengths of 4 μs were used.

##### Molecular Dynamics Simulations

Molecular dynamics simulations of the cecropin assembly inserted in aqueous solution were performed in GROMACS (v4.5.5) using the AMBER99SB-ILDN force field with 150 mm concentration of sodium and chloride molecules for charge neutralization. AMBER99SB-ILDN was chosen following testing of four force fields, and correctly reproduced phenomena were observed in the primary monomeric spectra whereas other force fields showed helical bias. Monomeric structures were simulated for 50 ns in the NPT ensemble following energy minimization, 200-ps equilibration in the NVT ensemble, and 200-ps equilibration in the NPT ensemble. The dimer structure was run for 100 ns with 200ips equilibration steps in the NVT and NPT ensembles, both with and without peptide position restraints. These extra steps allowed for relaxation of the solvent and the dimer in each ensemble. The initial helical configuration was obtained using the XPlor-NIH structure determination algorithm. In detail, the temperature was coupled using a velocity-rescaling thermostat (coupling time 0.1 ps) with separate peptide and solvent coupling groups. Pressure coupling was isotropic and achieved through a Parrinello-Rahman coupling scheme (coupling time 2.0 ps).

Bond lengths were constrained using the LINCS algorithm. The TIP3P water model was used, and a time step of 2 fs was used throughout. Coulomb and van der Waals forces were treated by a twin range cut-off scheme, with short range electrostatics experiencing a cutoff of 1 nm and long range by particle-mesh Ewald with grid spacing of 0.12 nm. Pair-lists were updated every five steps.

##### Minimum Inhibitory Concentration (MIC) Assay

MICs were determined by broth microdilution on *Pseudomonas aeruginosa* ATCC 27853, *Escherichia coli* K12, *Staphylococcus aureus* ATCC 25723, *Micrococcus luteus* NCIMB 13267, and *Bacillus subtilis* ATCC 6633 according to the Clinical and Laboratory Standards Institute. Typically, 100 μl of 0.5–1 × 10^6^ cfu/ml of each bacterium in Mueller-Hinton medium broth (Oxoid) was incubated in 96-well microtiter plates with 100 μl of serial 2-fold dilutions of the peptides (from 100 to 0 μm) at 37 °C on a three-dimensional orbital shaker. The absorbance was measured after peptide addition at 600 nm using a Victor 2 plate reader (PerkinElmer Life Sciences). MICs were defined as the lowest peptide concentration after 24 h at 37 °C. All tests were done in triplicate.

##### Stain-dead Antimicrobial Assay

*S. aureus (*ATCC 25723) culture (1 ml) was centrifuged to give a cell pellet, which was washed twice with 10 mm phosphate buffer, pH 7.4, before being reconstituted in phosphate buffer to give *A*_600 nm_ = 0.008. 1 ml of the solution was dispensed in a 2-well glass chamber (LabTek) with diluted (1/500) propidium iodide (1 mg/ml, from Invitrogen). The chambers with surface-settled bacteria (60 min) were mounted on a confocal microscope (Olympus) equipped with an incubation chamber at 37 °C. Propidium iodide fluorescence emission was monitored at 625 nm for 60 min (three frames/min) using an appropriate filter after the addition of peptide (1 ml). Recorded images (XYZ) were analyzed using Fiji software to plot the number of fluorescent (stain-dead) cells as a function of time.

##### Hemolysis Assay

Hemolysis was determined by incubating 10% (v/v) suspension of human erythrocytes with peptides. Erythrocytes were rinsed four times in 10 mm PBS, pH 7.2, by repeated centrifugation and resuspension (3 min at 3000 × *g*). Erythrocytes were incubated at room temperature for 1 h in either deionized water (fully hemolysed control), PBS, or with peptide in PBS. After centrifugation at 10,000 × *g* for 5 min, the supernatant was separated from the pellet and the absorbance measured at 550 nm. Absorbance of the suspension treated with deionized water defined complete hemolysis. The values below correspond to the percentage of hemolysis at tested concentrations. All tests were done in triplicate.

##### Gram Stain Assays

20 μl of a bacterium culture was dispensed onto a glass slide and spread well. The slide was swiftly passed through a Bunsen flame to dry and fix cells before staining. The fixed bacteria were first covered in crystal violet (0.25%) for 30 s followed by washing with water (distilled, filtered 0.22 μm) until all of the excess stain was washed off, and then with iodine (1.0%) for 2 min before washing with a minimum amount of acetone needed to rinse off the iodine color. The cells were counterstained with safranin (0.5%) for 30 s, washed with water, and dried by a Bunsen flame. The obtained slides were viewed under a Leica DMLB fluorescent microscope. The images were obtained at ×100 magnification under oil and analyzed by ImageJ.

## RESULTS

### 

#### 

##### Cathelicidin Assembly Design

b27 consists of 27 amino acid residues with one glycine at each terminus (20). The C-terminal heptad of the peptide is predominantly hydrophobic and contains a P*XX*P motif, which is believed to promote membrane insertion (22). A larger N-terminal stretch has three incomplete coiled-coil heptads starting with a charged arginine at *g* followed by putative *a*/*d* and *g*/*e* patterns. An antagonist sequence, anti-b27, was designed to match these patterns in a parallel arrangement. *a* and *d* positions were occupied by core-stabilizing combinations of phenylalanine and leucine residues to favor low oligomers while promoting cooperative folding (23–25). Negatively charged glutamates were used in *g* and *e* positions of anti-b27 to pair corresponding *g*′, *e*′ lysines and arginines in b27. The remaining *b*, *c*, and *f* sites were made neutral polar and alanine residues. The P*XX*P motif in the antagonist was deemed redundant and was replaced to stabilize coiled-coil formation. Fig. 1*A* summarizes the design.

##### Cathelicidin Assembly Folding

**FIGURE 2.**
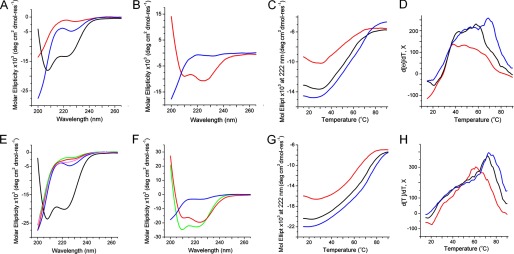
**Peptide folding probed by CD spectroscopy.** *A*, CD spectra for b27 (*red line*), anti-b27 (*blue line*), and anti-b27:b27 (*black line*) in 10 mm phosphate buffer. *B*, CD spectra for b27 (*red line*) and anti-b27 (*blue line*) in anionic membranes. *C* and *D*, thermal unfolding curves (*C*) and their first derivatives for anti-b27:b27 (*D*) as a function of temperature at 222 nm. *E*, CD spectra for cB (*green line*), cBt (*red line*), anti-cBt (*blue line*), anti-cBt:cB (*magenta line*), and anti-cBt:cBt (*black line*) in 10 mm phosphate buffer. *F*, CD spectra for cB (*green line*), cBt (*red line*), and anti-cBt (*blue line*) in anionic membranes. *G* and *H*, thermal unfolding curves (*G*) and their first derivatives (*H*) for anti-cBt:cBt as a function of temperature at 222 nm. CD spectra are for 30 μm concentration of each peptide, pH 7.4, room temperature. Thermal unfolding curves are for 15 μm (*red line*), 30 μm (*black line*), and 50 μm (*blue line*) concentration of each peptide. Lipid-peptide ratio for CD spectra in anionic membranes was 100:1.

Consistent with the design, the peptides did not fold individually in aqueous solutions at micromolar concentrations. Circular dichroism (CD) spectra were characteristic of random coil or disordered conformations (Fig. 2*A*). Similarly, none of the peptides folded in the presence of zwitterionic unilamellar vesicles whose composition mimics that of mammalian membranes (supplemental Fig. S1). In contrast, CD spectra for equimolar peptide mixtures showed appreciable helical signals indicative of coiled-coil formation (Fig. 2*A*). The behavior of the peptides in anionic unilamellar vesicles, which mimicked microbial membranes, was consistent with the observations. Unlike anti-b27, which remained disordered, b27 underwent a coil-helix transition suggesting membrane binding (Fig. 2*B*).

The helix content in both cases did not exceed 35% (supplemental Fig. S1). The percentage of α-helix was estimated using the equation: −100([θ]_222_ + 3000)/33,000 (26). However, the thermal denaturation of pair mixtures gave sigmoidal unfolding curves indicative of cooperatively folded structures (Fig. 2*C*). Low temperature CD and FTIR spectra recorded before and after thermal unfolding revealed almost complete reversibility of folding (supplemental Fig. S1). The first derivatives of the unfolding curves comprised several overlapping transitions with two clear cut-offs of transition midpoints (*T*_*m*_) at approximately 37 °C and 70 °C, with the higher *T*_*m*_ becoming more pronounced at higher concentrations (Fig. 2*D*). The transitions were broadly within the biokinetic temperature ranges. However, the lower cut-off remained apparent at all concentrations and was dominating at lower concentrations (Fig. 2*D*). Although b27 was strongly antimicrobial (Table 1) the effect of the antagonist on the activity of b27 could not be measured with certainty (results not shown). Therefore, anti-b27-b27 interactions were deemed insufficiently stable under physiological conditions. The lack of stability can be attributed to that *g-e*′ interactions available in b27 can support the assembly of only two combined heptads, which falls short of a stable coiled coil formed by three contiguous heptads (Fig. 1*A*) (11). The longer cB sequence was thus probed.

**TABLE 1:** **Biological activity of peptides**

Peptide	Minimum inhibitory concentration (MIC)					HE (LC_50_)*^a^*
	*E. coli* (K12)	*P. aeruginosa* (ATCC27853)	*B. subtilis* (ATCC6633)	*S. aureus* (ATCC6538)	*M. luteus* (NCIMB 13267)	
	μ*m*	μ*m*	μ*m*	μ*m*	μ*m*	μ*m*
b27	3.13 ± 0.43	4.51 ± 1.56	0.78 ± 0.01	>100	0.74 ± 0.33	≫250
Anti-b27	>200	>200	>200	>200	>200	≫250
cB	0.62 ± 0.01	1.56 ± 0.01	50.7 ± 0.10	>100	0.46 ± 0.20	≫250
cBt	1.28 ± 0.04	1.55 ± 0.01	6.33 ± 0.12	25.9 ± 0.06	0.36 ± 0.10	≫250
Anti-cBt	>200	>200	>200	>200	>200	≫250

*^a^* Human erythrocytes, 50% cell death compared with untreated cells, 1–9% at 250 μm.

##### Cecropin Assembly Design

cB comprises 35 amino acid residues and three G(*X*)_*n*_G motifs, which in principle can span five canonical coiled-coil repeats. However, all three G(*X*)_*n*_G motifs form a contiguous stretch starting in the second heptad from the N terminus which makes the assigning of contiguous coiled-coil patterns problematic. Searches using coiled-coil prediction algorithms (Paircoil2, COILS, Multicoil), albeit biased toward stand-alone sequences, did not give any apparent pattern either. Therefore, selective point mutations were allowed.

The central G(*X*)_4_GP motif and the tryptophan residue, important for activity (27), were kept in the sequence. The N-terminal lysine was assigned to the first *g* position. This ensured minimum mutations by placing isoleucines and leucines in *a* and *d* and by swapping hydrophobic and polar residues in *g* and *a* to enable contiguous *a-d*/*e-g* pairs (23–25). A single Ala→Lys mutation at an *e* position was adapted to enable a continuous network of electrostatic interhelical interactions, whereas a Glu→Gln mutation at a *b* position excluded an intrahelical *i, i*+4 electrostatic interaction. This completes a cB template, cBt. Similar to the anti-b27, anti-cBt was designed to mirror the *a-d*/*e-g* arrangements in cBt. Glutamates were used at all *e* and *g* sites, whereas isoleucines and leucines occupied *a* and *d* sites. The G(*X*)_4_GP was simplified to G(*X*)_5_P. This stretch is functionally irrelevant for anti-cBt, which is meant to be biologically inactive, but structurally it is sufficient to prevent the sequence of such length to fold autonomously. In the resulting anti-cBt:cBt arrangement all five heptads of cBt contribute to the binding with anti-cBt with the number of *e-g*′ pairs being nearly doubled compared with b27 (Fig. 1*B*).

##### Cecropin Assembly Folding

**FIGURE 3.**
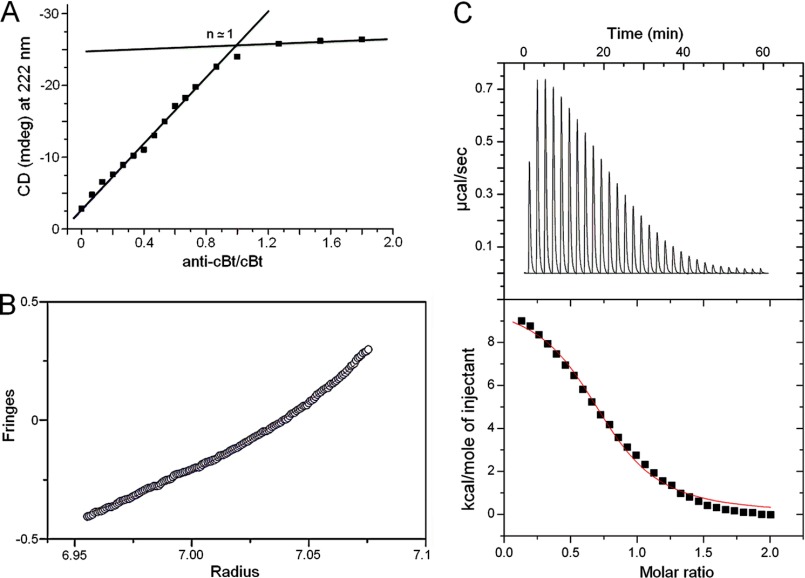
**Stoichiometry of anti-cBt:cBt interactions.** *A*, CD points recorded for 222 nm by titrating anti-cBt into cBt (30 μm). The *intersection* of the *lines* fitted on the titration curve indicates a 1:1 binding stoichiometry. *B*, sedimentation equilibrium analysis. Experimental data (*open circles*) were collected at 37,000 rpm for a 100 μm sample at 20 °C. The *line* is a calculated curve for an ideal dimer. *C*, isothermal titration calorimetry of the interactions. Heat absorbed (μcal/s) for each isotherm is plotted *versus* titration time (min) and shows endothermic binding (*top panel*). Integrated heats (kcal/mol) are plotted *versus* anti-cBt:cBt molar ratios (*bottom panel*).

The CD spectra of the pair in aqueous solutions revealed 20% increases in helicity (26) *versus* those recorded for anti-b27:b27 (Fig. 2, *A* and *E*, and supplemental Fig. S1). Comparable helical signals were recorded for cBt and cB in anionic membranes (Fig. 2*F*). None of the peptides folded in zwitterionic membranes (supplemental Fig. S1). CD spectra for individual peptides and the equimolar mixtures of cB with anti-cBt in solution as well as anti-cBt in anionic membranes were characteristic of random coils (Fig. 2, *E* and *F*). CD titrations performed by adding anti-cBt into the buffered cBt, which was kept at a constant concentration, provided quantitative information of coiled-coil interactions. The CD values (millidegrees at 222 nm) showed saturation after reaching an equimolar anti-cBt:cBt ratio (*n* = 1) allowing thus the stoichiometry of the complex being calculated from the titration curve as 1:1 (Fig. 3*A*). Sedimentation equilibrium data, which fitted an analysis that assumed a single ideal species, returned a *M*_r_ of 7148 (93% confidence limits of 6766 and 7527) for the 1:1 anti-cBt:cBt mixture, which was close to that expected for an anti-cBt:cBt dimer (7811), *versus* a *M*_r_ of 3508 (3328, 3691) for the monomeric anti-cBt (3843) (Fig. 3*B*). Further evidence comes from isothermal titration calorimetry experiments. Binding isotherms obtained for cBt titrated with anti-cBt support endothermic binding characteristic of native coiled-coil systems (28), and the integrated heats fitted into a single site binding model gave a stoichiometry of 1:1 (*n* = 0.8) and a strong association (binding) constant (*K*_*a*_) of 2.8 ± 0.2 × 10^6^ m^−1^. The endothermic binding enthalpy (Δ*H*) for this interaction was 9.7 kcal/mol with a favorable Δ*G* of −7.8 kcal/mol (Fig. 3*C*).

Consistent with the design, the results are comparable with those for other native and designed coiled-coil systems (28–30) and indicate that cB and cBt efficiently and selectively bind to anionic membranes and anti-cBt bundles up with cBt. Collectively, the data confirm cooperative and strong anti-cBt-cBt interactions, which remain at equilibrium with cBt.

**FIGURE 4.**
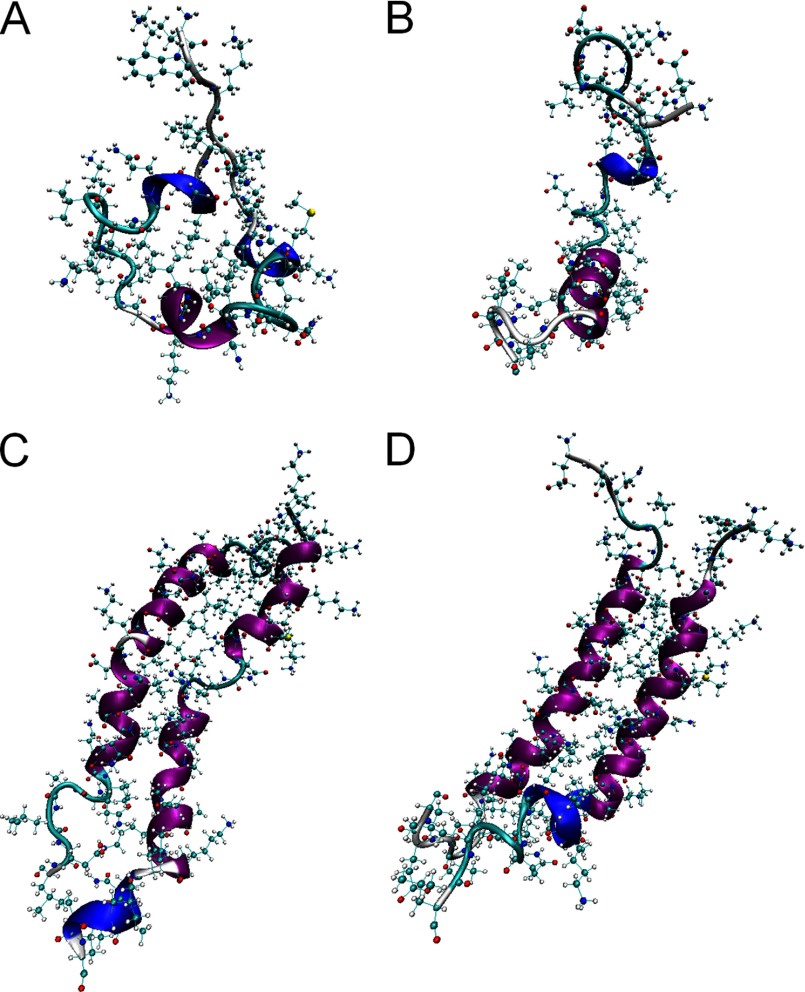
**Molecular dynamics simulations of cecropin assembly.** Secondary structure visualization after 50 ns for cBt (*A*) and anti-cBt (*B*), and for anti-cBt:cBt (*C*) at the initial configuration (0 ns) and after 100 ns (*D*).

To gain a better insight into oligomerization we performed molecular dynamics simulations using the Amber99SB-ILDN force field (supplemental text) (31). The 20-ns simulations of individual peptides revealed random conformations with no further significant changes to the peptides observed into 50 ns of simulations. Elements of helical structure in individual residues and short peptide stretches were present in both peptides which can be accounted for by their high helical propensities (Fig. 4, *A* and *B*, and supplemental Fig. S2). When visualized together following an equilibration phase, anti-cBt and cBt appeared to align in parallel supported by hydrophobic (*a-d*) and charged (*e-g*) contacts (Fig. 4*C*). At initial configurations (0 ns) the C-terminal fragments and hinge regions proved to be partly unfolded, but over 100 ns the assembly evolved into a rigid α-helical structure (Fig. 4*D*), which was consistent with the secondary structure analysis of each residue using the STRIDE secondary structure algorithm (supplemental Fig. S2). The C-terminal fragments retained a degree of unfolding, suggesting some fraying of the assembly. This arrangement was maintained over 100 ns conforming to the formation of a stable and cooperatively folded structure.

The findings were further supported by sigmoidal unfolding curves of anti-cBt:cBt (Fig. 2*G*). The first derivatives of the curves gave dominating transition midpoints at >60 °C, which at higher concentrations increased to >70 °C (Fig. 2*H*). The obtained spectra had some structure suggesting partial unfolding, consistent with the simulations, or α-to-β conformational transitions. CD spectra recorded after the thermal denaturation agreed with incomplete reversibility of folding, whereas FTIR spectra recorded before and after thermal denaturation showed nearly identical helical bands at 1650 cm^−1^ and 1550 cm^−1^, with no indication of a β-sheet switch (1610–1625 cm^−1^) (supplemental Fig. S1). CD spectroscopy can only reveal averaged or relative changes in helicity corresponding to mixed types of binding comprising membrane-bound antimicrobial peptides and assembled coiled coils, both of which are associated with helix formation.

**FIGURE 5.**
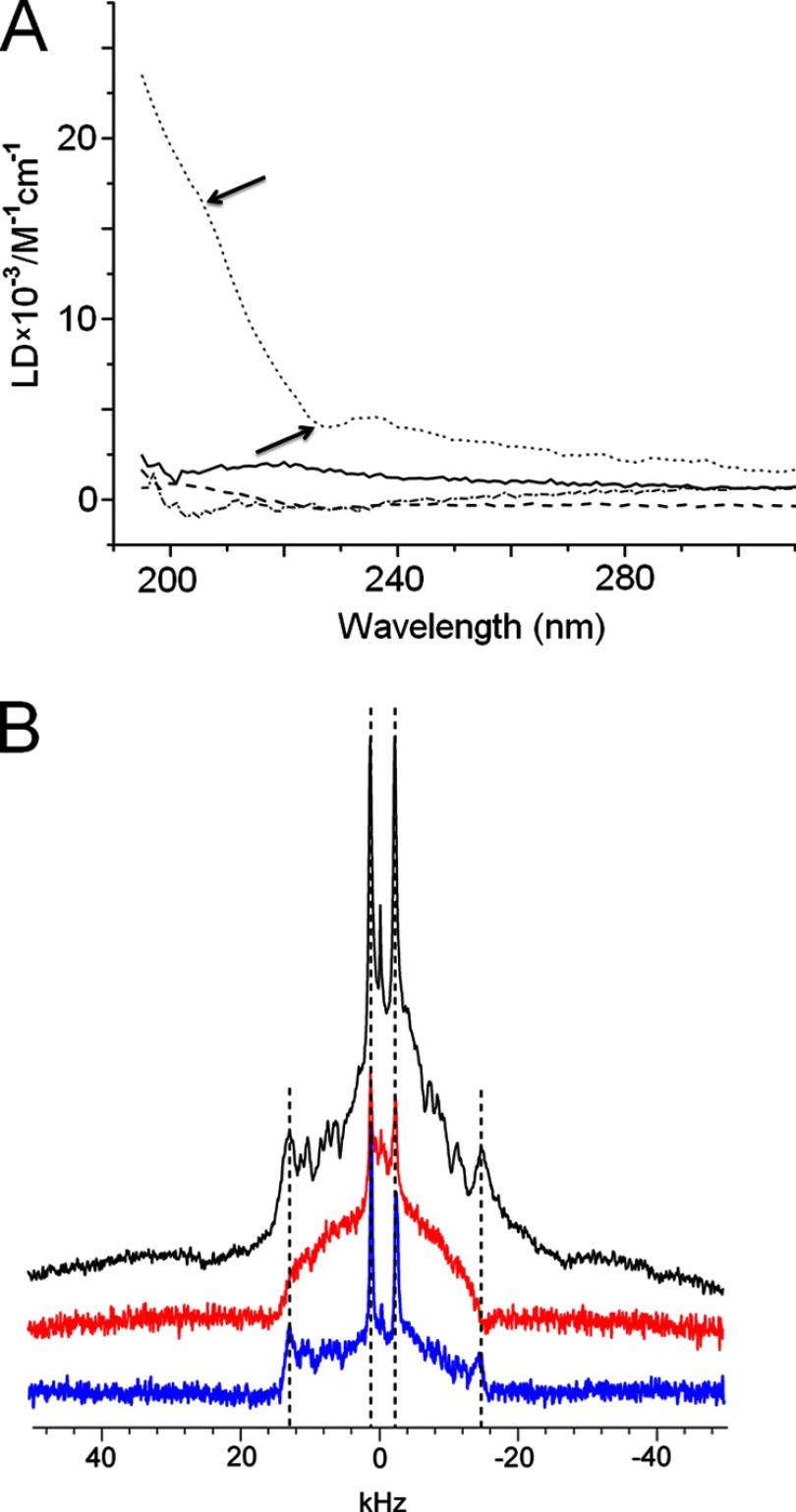
**Cecropin folding in anionic membranes.** *A*, LD spectra for cBt (*dotted line*), anti-cBt (*bold line*), cBt added to anti-cBt (*dashed line*) and pre-formed anti-cBt:cBt (*dot-dashed line*). Lipid-peptide ratio was 100:1 (20 μm peptide), pH 7.4, room temperature. *Arrows* point to π-π* and n-π* electronic transition bands. *B*, solid-state NMR spectra for blank (no peptide, *black*), cBt (*red*), and anti-cBt:cBt (*blue*). Lipid-peptide ratio was 25:1, pH 7.4, room temperature. *Vertical dashed lines* are to assist comparison between the innermost and outermost splittings of the three spectra.

Further evidence for antagonist effects on antimicrobial peptides may be ascertained from changes in membrane binding monitored by LD spectroscopy. LD arises from differential absorption of light linearly polarized parallel and perpendicular to an orientation axis and thereby provides a convenient probe of relative peptide orientation to the membrane surface (32). LD spectra for cB and cBt in anionic membranes were similar and showed positive and negative bands at approximately 200–210 nm and 220–230 nm, respectively, which are characteristic of π-π* and n-π* electronic transitions of peptide helices lying flat on membrane surfaces (33). This is consistent with the proposed carpet-like mechanism of action for cB (34) and indicates that cBt should retain this mode of action (Fig. 5*A* and supplemental Fig. S3).

Negligible signals were recorded for anti-cBt in anionic membranes (Fig. 5*A* and supplemental Fig. S3) and for all peptides in zwitterionic membranes (supplemental Fig. S3). In the presence of anti-cBt no absorption patterns could be identified, suggesting interfering contributions from anti-cBt (Fig. 5*A* and supplemental Fig. S3). LD spectra for preformed anti-cBt:cBt complexes at different ratios were also negligible without specific binding or orientation, but revealed shape similarities with the spectra for cBt implying competing interactions of cBt for anti-cBt and membrane surfaces (Fig. 5*A* and supplemental Fig. S3). Further support for this conclusion comes from solid-state NMR studies.

Fig. 5*B* shows static ^2^H spectra of anionic membranes with chain-deuterated lipids (DMPC-d54) in the absence and presence of the peptides. Spectra for the membrane alone showed maximum and minimum splittings of 28 and 4.9 kHz, respectively. Intermediate peaks that correspond to CD_2_ groups along the fatty acid acyl chains were well resolved. On addition of cBt, no individual peaks could be resolved, suggesting that the peptide interacts with and disorders the lipid molecules along the length of the acyl chains. In marked contrast, the innermost and outermost splittings for anti-cBt:cBt were consistent with the lipid only spectrum, suggesting that anti-cBt prevents cBt from disrupting the lipids. In all of the spectra, the innermost splitting corresponding to the CD_3_ groups on the ends of the acyl chains was apparent, implying that the lipid termini remained buried within the lipid bilayer, which together with that there were no dominant peaks observed at 0 kHz suggests that micellization did not take place (Fig. 5*B*). The observations are consistent with the CD and LD data in that cBt folds onto anionic membranes and binds with its antagonist through cooperative interactions that interfere with membrane binding (35).

##### Cecropin Assembly in Bacterial Culture

The results discussed so far support the formation of anti-cBt:cBt complexes that can exist in dynamic membrane environments. However, to be competitive under equilibrium conditions the interactions are likely to require anti-cBt in excess. In physiological terms, this is consistent with that AMPs must respond to microbial challenge within their proteolytic life time (*i.e.* minutes) and that inhibitory effects on antimicrobial activity should become apparent within the same time scale.

**FIGURE 6.**
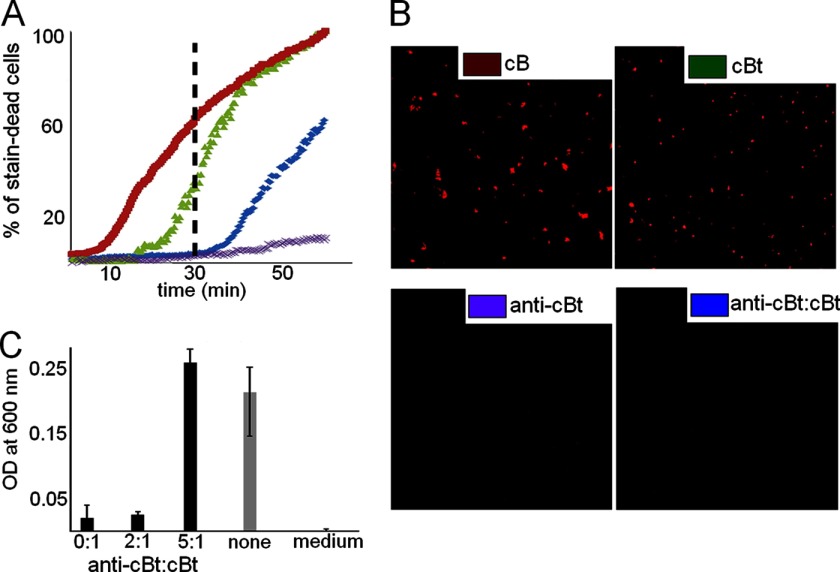
**Stain-dead *S. aureus* cells.** *A*, percentage of stain-dead cells as a function of time for cB (100 μm, *red squares*), cBt (25 μm, *green triangles*), anti-cBt (250 μm, *purple crosses*), and anti-cBt:cBt (125:25 μm, *blue diamonds*). *B*, confocal microscopy images of cells stained with propidium iodide after incubation with corresponding peptide. *C*, absorbances measured overnight at 600 nm for the bacterium incubated with anti-cBt:cBt at different ratios (*black pillars*), for the bacterium without peptide and for the culture medium only (*gray pillars*). *Error bars*, S.D.

To probe this, we performed stain-dead assays using a log-phase planktonic culture of *S. aureus*. MICs against the bacterium were at 15–30 μm ranges (25 μm, Table 1), at which anti-cBt:cBt were stable up to 70 °C (Fig. 2*H*). In these assays the fluorescence emission of propidium iodide used as a dead cell marker was measured after peptide addition as a function of time (Fig. 6).

**FIGURE 7.**
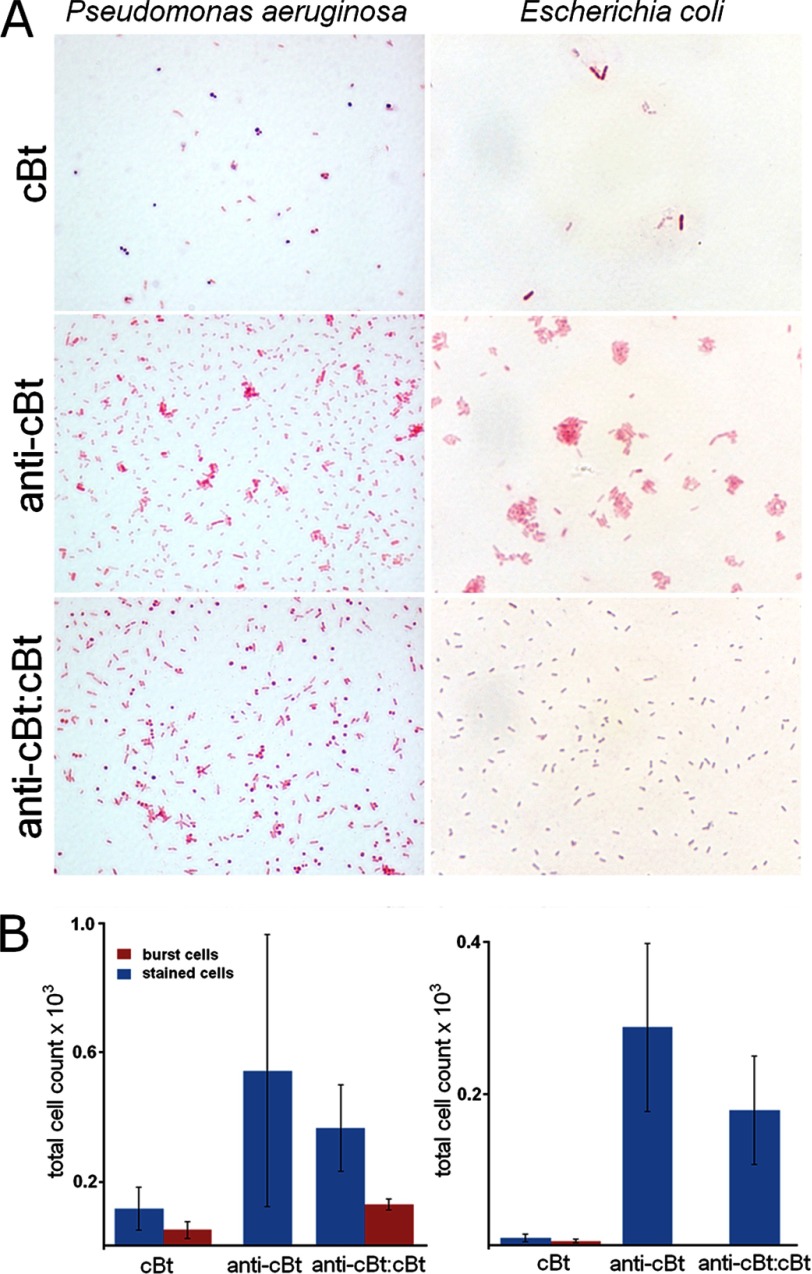
*A*, 100× light micrographs of Gram-stained *E. coli* and *P. aeruginosa* after overnight incubations with peptide. *B*, averaged total of Gram-stained cells. Shown is anti-cBt:cBt at (100:10 μm).

To directly assess the inhibition of cBt by anti-cBt the total number of cells lysed by cBt at the MIC after 1 h was taken as 100% killing rates. Thus, in the first 30 min killing rates for cB and cBt were ∼60 and ∼40%, respectively (Fig. 6, *A* and *B*). No effects observed for anti-cBt at concentrations >100 μm, and marginal killing rates (10%) at higher concentrations (>250 μm) were consistent with the medium cytotoxicity of the peptide at hemolytic concentrations (Table 1). For anti-cBt:cBt at 5–10:1 molar ratios a continuous lag-phase in killing rates of <10% was observed over the first 40 min of incubation (Fig. 6, *A* and *B*). The effect then became less apparent with gradual exponential increases in killing rates up to 60% after 60 min accounting for 40% decreases in the activity of cBt. The optical density measurements of bulk culture overnight (MIC assays) at 5–10:1 ratios gave the visible growth of *S. aureus* which was comparable with that of the bacterium without peptide (Fig. 6*C*). In contrast, lower anti-cBt:cBt ratios (<5:1) imposed no inhibition on the antimicrobial activity of cBt (Fig. 5*C*). *E. coli* and *P. aeruginosa* that were found to be more susceptible to cBt (Table 1) gave varied and irreproducible MIC differences between cBt and anti-cBt:cBt. Because optical density measurements take no account of changes at the cellular level we sought additional evidence from Gram stain assays for these strains. *E. coli* and *P. aeruginosa* incubated with peptide for 16 h were Gram-stained and imaged by optical microscopy (Fig. 7*A* and supplemental Fig. S4). Nearly complete inhibition of cell growth by cBt was apparent for both strains. No obvious effects were found for anti-cBt and anti-cBt:cBt samples (10:1) in which cell populations were remarkably similar for both strains (Fig. 7, *A* and *B*). Round and blue-stained spheroplasts, particularly for *P. aeruginosa*, were observed for cBt and anti-cBt:cBt samples, but not in anti-cBt samples. This is consistent with spheroplast-forming activities of cell wall or membrane-active antibiotics (36), which implies that anti-cBt:cBt may retain residual antimicrobial activity of cBt giving rise to cell wall deficient-forms (Fig. 7).

## DISCUSSION

The results prompt several conclusions. Firstly, anti-antimicrobial activity may occur at a microscopic level, which is not necessarily detected by changes in optical density. Second, the 5–10:1 ratios of anti-AMP to AMP give detectable anti-antimicrobial effects. Third, coiled-coil stability, which directly links to primary structure, determines all of these points. Indeed, other antimicrobial sequences that are biologically and structurally similar to cB and b27 are shorter. This decreases the probability of shorter antagonists and their assemblies with AMPs. Magainin 2 (m2) can serve as an example. The peptide is a 23-mer with one G(*X*)_4_G motif, which thus can represent a shorter version of cB. Although it was possible to assign a coiled-coil template in the sequence our attempts to find an efficient antagonist were unsuccessful (supplemental Figs. S5 and S6). Furthermore, mutations used to generate suitable magainin templates significantly compromised membrane binding (supplemental Fig. S6). This lack in sequence plasticity may not come as a surprise if one considers that shorter antimicrobial helices may aim at minimizing the possibility of co-folding with potential antagonists, which in turn are challenged to produce stronger binders. Such a competition is pre-defined in native AMPs which contain the patterns of both antimicrobial helices and coiled coils. The former is thus designed to prevail in membrane environments but can become “frustrated” by a competitive trigger, an antagonist, which gives way to the latter.

Antimicrobial peptides are being considered as new drug candidates in the post-antibiotic era (7). Their clinical potential is largely attributed to that widespread microbial resistance against them has yet to emerge (10, 37, 38). In this context, it is plausible to suggest that antagonistic sequences secreted by bacterial cells or expressed on their surfaces may elicit anti-antimicrobial responses. Although this remains to be shown in nature, this report, to the best of our knowledge, provides the first example of peptide sequences capable of antagonistic responses to the action of host defense peptides. Specifically, the combination of experimental and computational approaches confirms a coiled-coil-mediated antagonistic mechanism which is consistent with the functional basis of antimicrobial peptides as local and short contact time immune regulators.

## Supplementary Material

Supplemental Data

## Footnotes

2 The abbreviations used are:

AMP: antimicrobial peptide
DIPEA: *N*,*N*-diisopropylethylamine
DLPC: 1,2-dilauroylphosphatidylcholine
DLPG: 1,2-dilauroyl-*sn*-glycero-3-phospho-(1′-rac-glycerol)
DMPC: 1,2-dimyristoyl-*d*54-*sn*-glycero-3-phosphocholine
DMPG: 1,2-dimyristoyl-*sn*-glycero-3-phospho-(1′-rac-glycerol)
HCTU: 2-(6-chloro-1H-benzotriazole-1-yl)-1,1,3,3-tetramethylaminium hexafluorophosphate
LD: linear dichroism
MIC: minimum inhibitory concentration.
