# Supplementary material for: Anti-antimicrobial Peptides: FOLDING-MEDIATED HOST DEFENSE ANTAGONISTS
Source: J Biol Chem. 2013 Jun 4;288(28):20162–72. doi: 10.1074/jbc.M113.459560 (PMC3711284; doi:10.1074/jbc.M113.459560)
Supplement: Supplemental Data [file supp_288_28_20162__index.html]

Anti-antimicrobial peptides: folding-mediated host defense antagonists — Anti-antimicrobial Peptides — Anti-antimicrobial Peptides — Supplemental Data 

# Anti-antimicrobial Peptides

## 

**Files in this Data Supplement:**

- Supplemental Figures 1-6 (.pdf, 2.4 MB) - Figure S1. Peptide folding probed by CD and FTIR spectroscopy. Figure S2. Molecular dynamics simulations of the cecropin assembly type. Figure S3. LD spectra. Figure S4. 100x light micrographs of Gram-stained P. aeruginosa and E. coli Figure S5. Peptide design for magainin 2 type. Figure S6. Peptide (magainin) folding probed by CD spectroscopy.
