# Supplementary material for: Anti-antimicrobial Peptides: FOLDING-MEDIATED HOST DEFENSE ANTAGONISTS
Source: J Biol Chem. 2013 Jun 4;288(28):20162–72. doi: 10.1074/jbc.M113.459560 (PMC3711284; doi:10.1074/jbc.M113.459560)
Supplement: Supplemental Data [file supp_M113.459560_jbc.M113.459560-1.pdf]

Anti-antimicrobial peptides: folding-mediated host defense antagonists

Lloyd Ryan, Baptiste Lamarre, Ting Diu, Jascindra Ravi, Peter J. Judge, Adam Temple, Matthew Carr, Eleonora Cerasoli, Bo Su, Howard F. Jenkinson, Glenn Martyna, Jason Crain, Anthony Watts, and Maxim G. Ryadnov

Supplemental Data

Magainin assembly type. In m2, a plausible *a-d*/*e-g* pattern starts with the *N*-terminal lysine at *g*. The preceding GIG motif, which does not contribute to helix formation but breaks it, was avoided. Ile could serve as an *a* site, where it is favoured in coiled-coil dimers. However, in allocating *e-g* patterns it was important to maximize the use of native lysine residues avoiding extensive mutations, which otherwise was not straightforward. The lysine favours the combination of phenylalanine in *a* with a SAKK cluster for *defg*. With a hydrophobic leucine replacing serine the phenylalanine can provide stabilizing *a-a′* and *d-d′* interfaces with an antagonist, which together with another lysine in place of alanine completes the first heptad with an *e* site (Figure S5). A second heptad, with a single Phe→Lys mutation allowed to set up lysines at the following *g* and *e* positions, was complete with native phenylalanine and alanine residues stabilizing at *a-d*. A subsequent and third heptad required substantial mutations. Therefore, the seven C-terminal residues were deemed redundant for coiled-coil formation and left unchanged with the exception of a Glu→Gln mutation to exclude a competing *i, i+3* interaction with the introduced lysine, and a Met→Gln mutation for synthetic reasons. Taken together this gave rise to a putative coiled-coil template, m2t. An antagonist sequence was copied on the pattern with the lysines at *e* and *g* positions converted into glutamates to allow electrostatic interactions with m2t. The GxxxxG stretch was retained in both sequences. The stretch is helix-destabilising the effect of which may not be compensated by a two-heptad overlap in m2t which falls short of three heptads necessary for stable coiled-coil formation. Alternative patterns for m2t were possible, but none could provide a two-heptad overlap. Three-heptad overlap was generated in m2t2, which required additional mutations – phenylalanines were replaced by isoleucines and an additional Ala→Leu mutation was introduced to mimic a leucine zipper arrangement. Lysine mutations were made in the C-terminal heptad to introduce another pair of *e-g′* interactions (Figure S5).

Figures

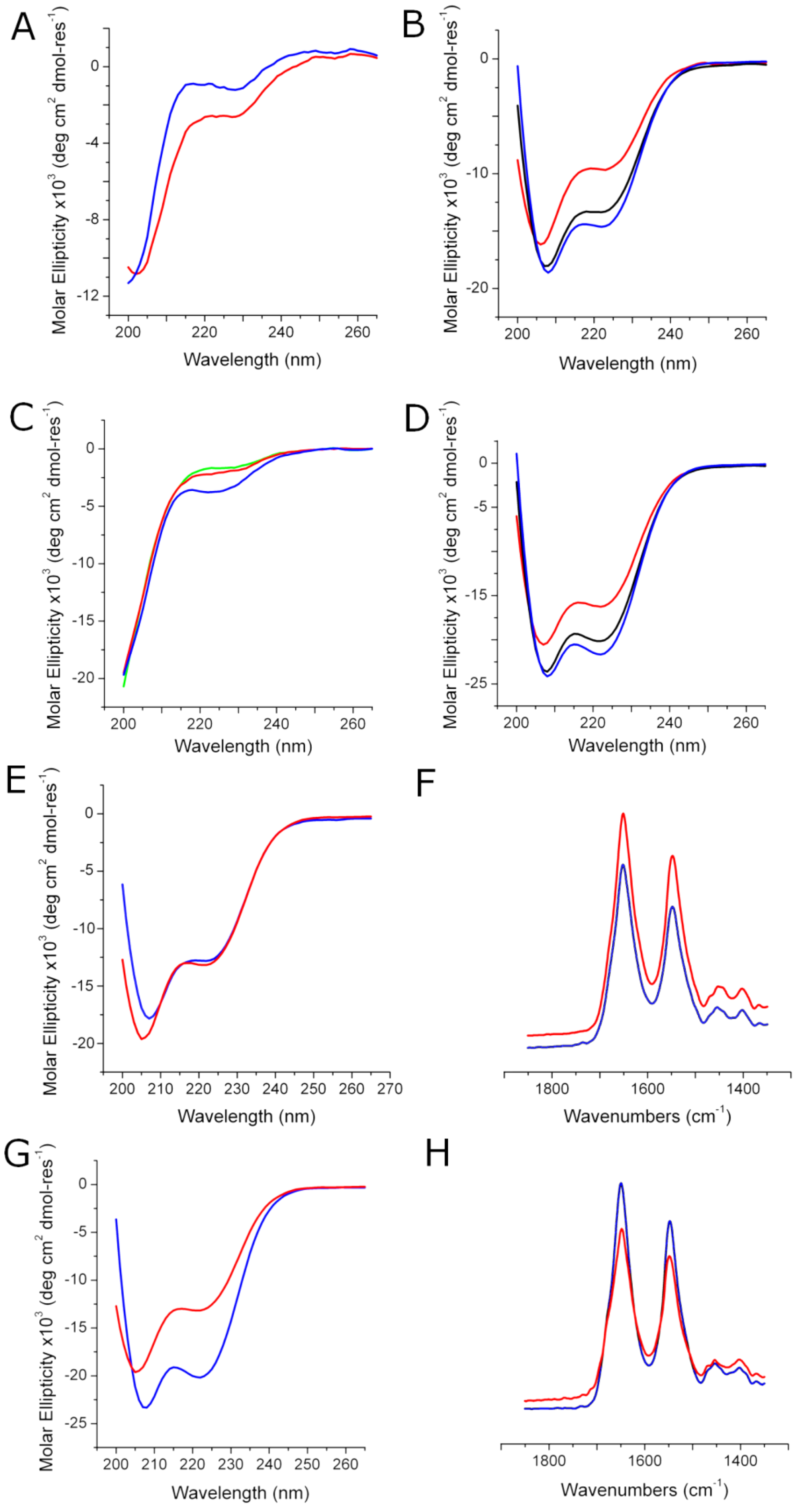

Figure S1. Peptide folding probed by CD and FTIR spectroscopy. CD spectra for (A) anti-b27 (blue line) and b27 (red line) in zwitterionic membranes, (B) anti-b27:b27 for 15 µM (red line), 30 µM (black line) and 50 µM (blue line) in each peptide, (C) anti-cBt (blue line), cBt (red line) and cB (green line) in zwitterionic

membranes at 30 μM in each peptide, (D) anti-cBt:cBt for 15 μM (red line), 30 μM (black line) and 50 μM (blue line) in each peptide. CD (E, G) and FTIR (F, H) spectra before (blue) and after (red) thermal denaturation for anti-b27:b27 (E, F) and for anti-cBt-:cBt (G, H).

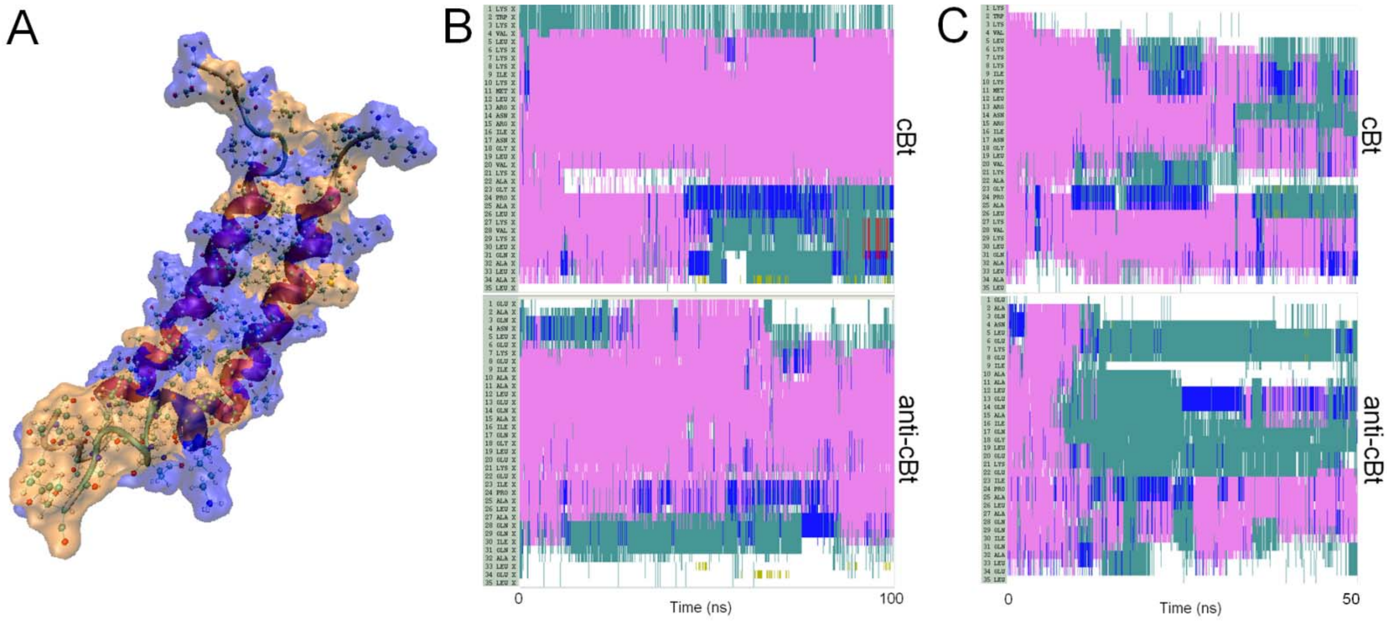

Figure S2. Molecular dynamics simulations of the cecropin assembly type. (A) Secondary structure visualization of anti-cBt:cBt after 100 ns with hydrophobic and electrostatic interfaces shown in orange and blue, respectively. (B) Secondary structure of each residue as a function of time for anti-cBt and cBt. Key: pink is for α-helix, blue for 3_{10}-helix, yellow for β-structure, green for turn and white for unordered.

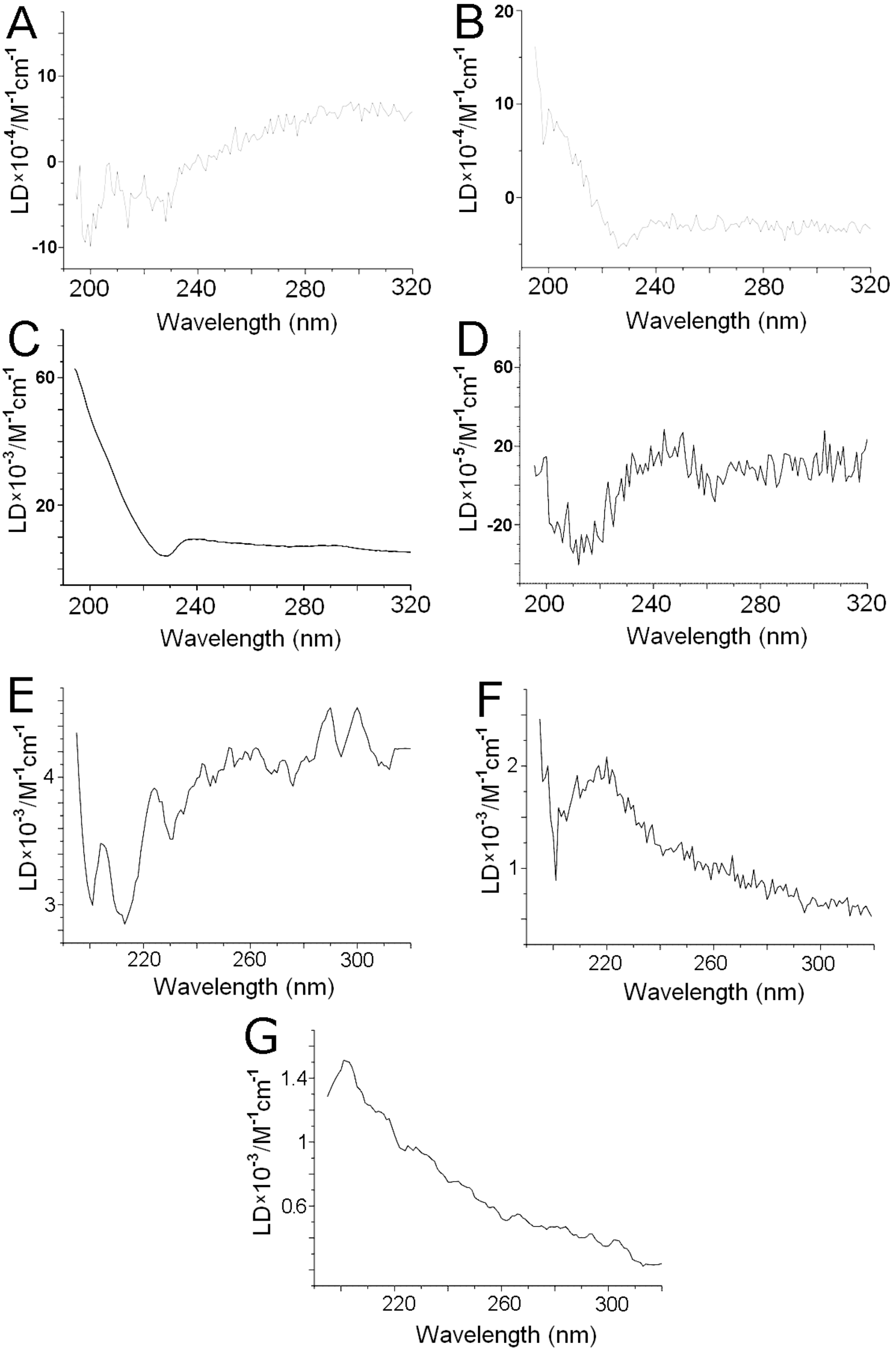

Figure S3. LD spectra in anionic (A-C) and zwitterionic (E-G) membranes for (A) cBt added to anti-cBt, (B) pre-formed anti-cBt:cBt, (C) cB and (D) anti-cBt, (E) cBt, (F) anti-cBt, (G) cB. Lipid-peptide ratio 100:1 (20 μM peptide), pH7.4, room temperature. Note should be taken to the differences in LD intensities for A, B (10^{-4}), D (10^{-5}) and C, E-G (10^{-3}).

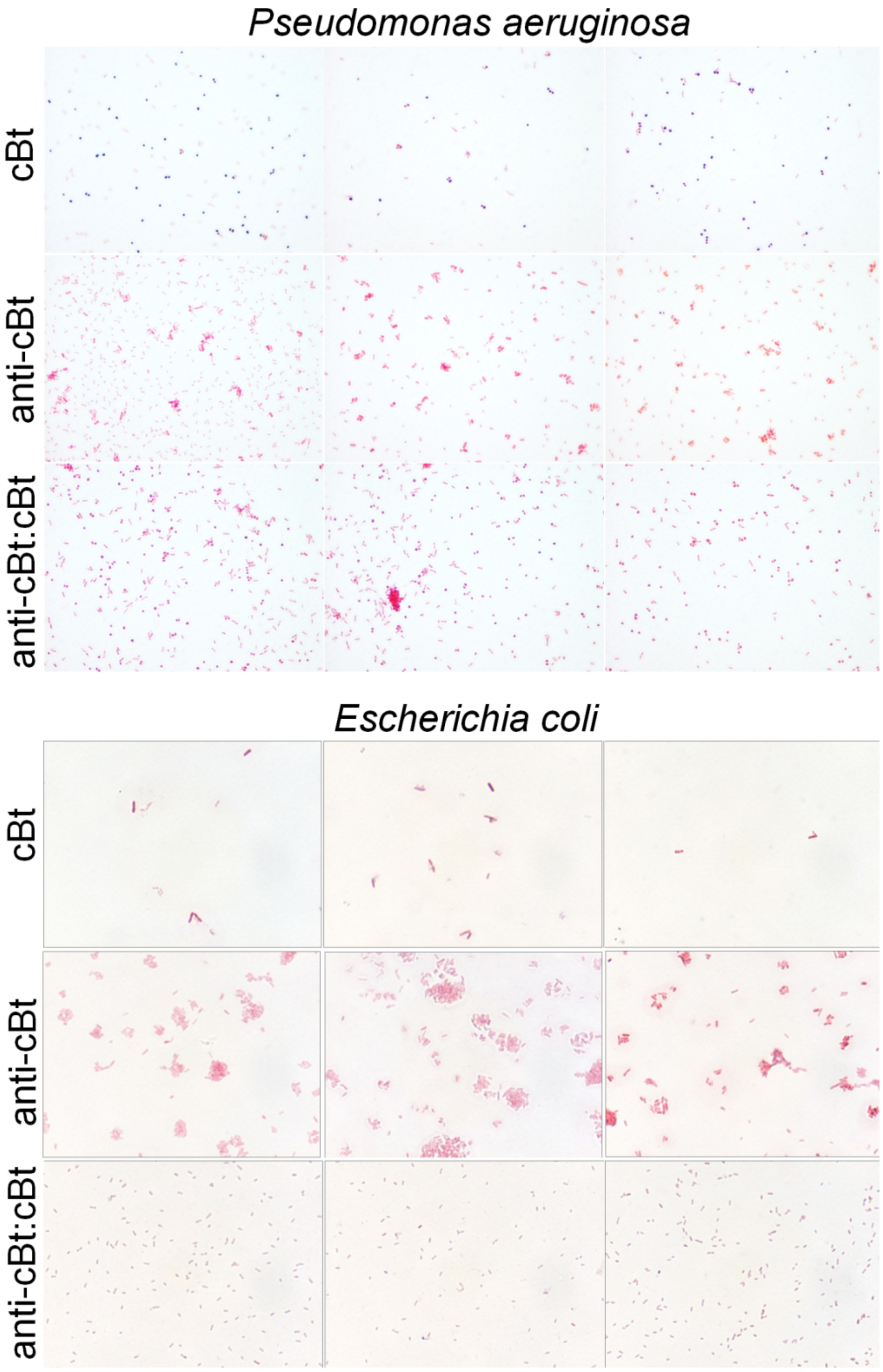

Figure S4. 100x light micrographs of Gram-stained *Pseudomonas aeruginosa* and *Escherichia coli* after overnight incubations with peptide. Key: anti-cBt:cBt at 100:10 μM.

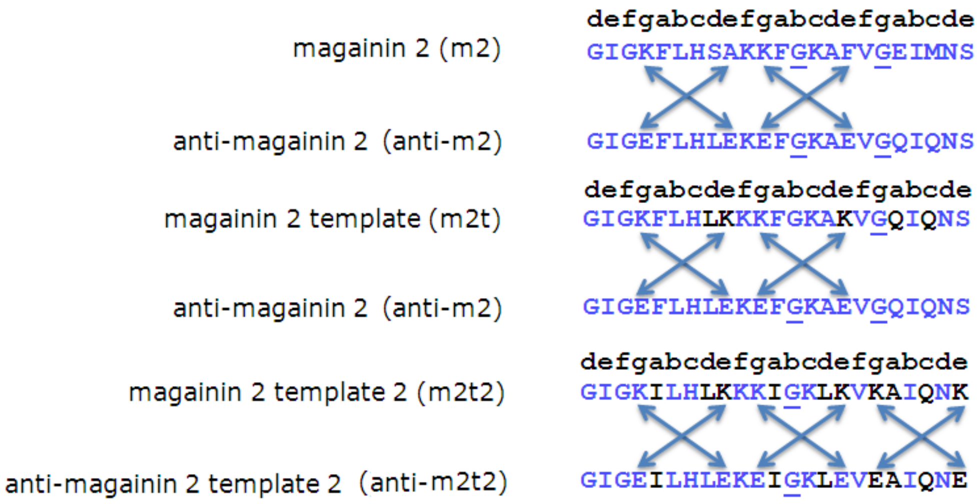

Figure S5. Peptide design for magainin 2 type. Native magainin 2 (m2), anti-magainin 2 (anti-m2), magainin 2 template (m2t), magainin 2 template 2 (m2t2), anti-magainin 2 template 2 (anti-m2t2). Linear sequences with mutations sown in black and the terminal residues of helix-disrupting motifs underlined. Putative electrostatic *e*/*g* interactions are shown by double-headed arrows.

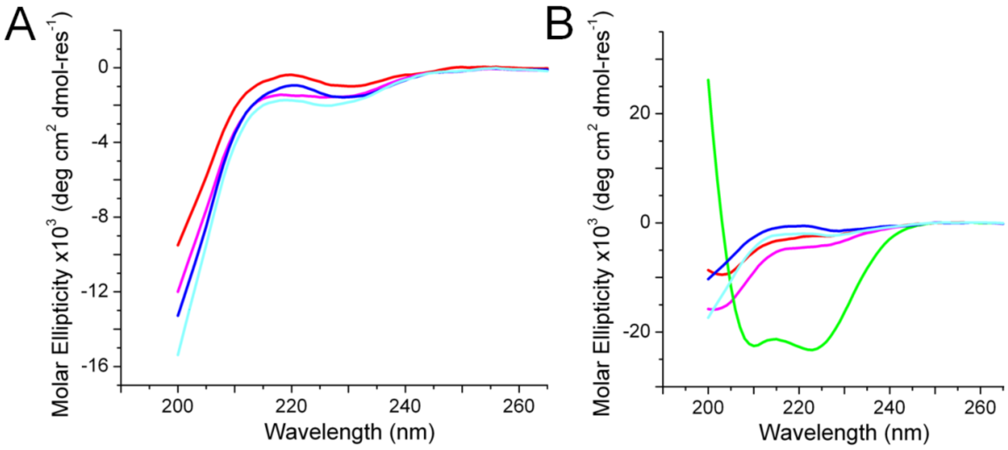

Figure S6. Peptide folding probed by CD spectroscopy. (A) CD spectra in 10 mM phosphate for anti-m2:m2 (blue line), anti-m2t:m2 (red line), anti-m2t2:m2t2 (magenta line) and anti-m2t2:m2 (cyan line). (B) CD spectra in anionic membranes for m2 (green line), m2t (red line), m2t2 (magenta line), anti-m2 (blue line), anti-m2t2 (cyan line). Spectra are for 30 μM in each peptide, pH 7.4, room temperature, lipid-peptide ratio 100:1.
